# A spectrum of clinical severity of recessive titinopathies in prenatal

**DOI:** 10.3389/fgene.2022.1064474

**Published:** 2023-01-25

**Authors:** Yiming Qi, Xueqi Ji, Hongke Ding, Yunan Wang, Xin Liu, Yan Zhang, Aihua Yin

**Affiliations:** ^1^ Prenatal Diagnosis Centre, Guangdong Women and Children Hospital, Guangzhou, China; ^2^ Maternal and Children Metabolic-Genetic Key Laboratory, Guangdong Women and Children Hospital, Guangzhou, China; ^3^ Guangzhou Medical University, Guangzhou, China; ^4^ Aegicare, Shenzhen, China

**Keywords:** TTN metatranscript-only, hydrops fetalis, arthrogryposis, prenatal diagnostics, titinopathies

## Abstract

Variants in TTN are associated with a broad range of clinical phenotypes, from dominant adult-onset dilated cardiomyopathy to recessive infantile-onset myopathy. However, few foetal cases have been reported for multiple reasons. Next-generation sequencing has facilitated the prenatal identification of a growing number of suspected titinopathy variants. We investigated six affected foetuses from three families, completed the intrauterine course of the serial phenotypic spectrum of TTN, and discussed the genotype-phenotype correlations from a broader perspective. The recognizable prenatal feature onset at the second trimester was started with reduced movement, then contracture 3–6 weeks later, followed with/without hydrops, finally at late pregnancy was accompanied with polyhydramnio (major) or oligohydramnios. Two cases with typical arthrogryposis-hydrops sequences identified a meta-only transcript variant c.36203-1G>T. Deleterious transcriptional consequences of the substitution were verified by minigene splicing analysis. Case 3 identified a homozygous splicing variant in the constitutively expressed Z-disc. It presented a milder phenotype than expected, which was presumably saved by the isoform of corons. A summary of the foetal-onset titinopathy cases implied that variants in TTN present with a series of signs and a spectrum of clinical severity, which followed the dosage/positional effect; the meta-only transcript allele involvement may be a prerequisite for the development of fatal hydrops.

## Introduction

The 363-exon *TTN* gene [TTN, OMIM (188840)] encodes titin, the largest-known protein. It bridges half of the entire sarcomere, is involved in the formation, provides passive stiffness and modulates active contractile force to the striated muscle sarcomere (Bang et al., 2001; Linke, 2018; Wang et al., 2021). The molecular architecture of the titin protein is organized into four distinct parts: the N-terminal Z-disc, the I-band and A-band regions, and the C-terminal M-line extremity encoded by the last six exons (358–363, or Mex1-6) (Gigli et al., 2016; Wang et al., 2021). Extensive *TTN* alternative splicing (AS) based on the presence of the N2A and N2B elements within the I-band generates numerous isoforms in different tissues and in different developmental and physiological states (Ottenheijm et al., 2009). Notably, the IC isoform (NM_001267550.1, inferred complete *TTN* meta transcript), which contains unique exons, is thought to be expressed only during embryonic development (Savarese et al., 2016; Savarese et al., 2018).

Mutations in the *TTN* gene cause a broad range of cardiomyopathies and/or skeletal muscle diseases, with autosomal dominant or recessive inheritance (LeWinter and Granzier, 2013; Ferreiro and Andoni Urtizberea, 2017). Monoallelic truncating variants in *TTN* (TTNtvs) predispose to dilated cardiomyopathy (DCM) in adults (LeWinter and Granzier, 2013). Atypical, more severe, prenatal or infant-onset titinopathies were associated with biallelic *TTN* variation, termed “congenital titinopathy” (Ferreiro and Andoni Urtizberea, 2017). Despite this, prenatal titinopathies have scarcely been depicted. In the largest cohort (n = 132) of recessive titinopathy to date, postnatal features are still the primary focus (Savarese et al., 2020a). Antenatal records of titinopathies were incoherent and fragmented. Possible reasons for the phenomenon are as follows:

1) Titinopathies are located at the borders of several groups of muscular pathologies with extremely high clinical heterogeneity (Perrin et al., 2020). The prenatal phenotype described in scattered studies is non-specific and concealed, and muscle tissue is difficult to collect in a foetus. *TTN* variants identified in a suspected foetus may overlap with another Mendelian disease with overlapping clinicopathological features. 2) Massive amounts of AS result in a remarkable diversity of titin isoforms (Guo et al., 2010). Isoform expression varies greatly not only in myocardium and skeletal muscle but also *in utero* and after birth (Walker and de Tombe, 2004; Ottenheijm et al., 2009; Savarese et al., 2018). It manifests as marked intrafamilial and interfamilial phenotypic heterogeneity in the affected foetus and adult. 3) The large symmetrical repetition region in *TTN* is frequently poorly analysed due to technical artefacts (Chauveau et al., 2014). Sheer size is also a problem, and rare variants in *TTN* are likely detected in any individual under extensive genetic screening (Savarese et al., 2020b; Miller et al., 2021). Unambiguous interpretation of these rare molecular findings, especially those detected accidentally without any features, is almost impossible in many foetuses. 4) The identification and characterization of the impact of specific regions of variation on phenotype is far from exhaustive. Patients with at least one pathogenic variant in the M-band resulting in a shorter, tailless protein may present a congenital phenotype (Savarese et al., 2020a). A truncation in the A-band domain of *TTN* perhaps causes DCM decades after birth (Hinson et al., 2015), whereas a truncation in the I band may be better tolerated because of in-frame deletion or AS (Perrin et al., 2020), and may prenatally present normal. The abovementioned reasons make it less possible to confidently predict *TTN* involvement when *TTNtvs* are unexpectedly identified *in utero*.

To facilitate prenatal diagnostic assessment and to better understand the comprehensive natural history of the antenatal subtype of titinopathy, we for the first time described in detail the prenatal course of six foetuses with biallelic *TTN* variants, thus, filling up the prenatal piece of the puzzle in titinopathies.

## Methods

### Participants

All pedigrees included in this study were the foetuses with identified causative variants of TTN. Patients with difficult-to-interpret missense variants of *TTN* were excluded to gain the clearest possible clinical picture of this disorder (Miller et al., 2021). Phenotypic data were collected by reviewing medical records, imaging, patient complaints, and clinical photographs. Chorionic villus sampling, amniocentesis or cordocentesis was executed strictly according to the guide of ISUOG (Cruz-Lemini et al., 2014).

### Whole-exome sequencing

Genomic DNA was extracted using standard protocols. Trio WES was performed using 2 × 150 bp in the paired end mode of the NextSeq 500 platform (Illumina, San Diego, CA) to obtain an average coverage of above 110x, with 97.6% of target bases covered at least 10x. All suspected variants were confirmed by Sanger sequencing.

### Minigene constructs containing a genomic

Fragments spanning from Exon167 (84 bp) to Exon169 (84 bp) of *TTN* were synthesized and cloned into the pcDNA3.1 plasmid. Mutation c.36203-1G>T was introduced with the QuickChange II XL site-directed mutagenesis kit (Agilent Technologies, Santa Clara, CA) according to the manufacturer’s instructions. Wild-type and mutant minigene constructs were transiently transfected into HeLa and HEK-293 cells, respectively, using Lipofectamine (Invitrogen, USA). Then, cells were incubated for 72 h before isolation of total RNA using Tiangen Reagent (Tiangen, Beijing, China). cDNA was synthesized using 2 μg of RNA with the MMLV reverse transcriptase kit (Promega, Madison, WI) and amplified with specific primers, including primers upstream in Exon 167 (forward 5-GCT​TGG​TAC​CAT​GGT​GCC​TGA​AGC​TCT​CCA​AGA​A-3) and downstream in Exon 169 (reverse 5-TCG​AGC​GGC​CGC​CAC​TTT​AAC​AGG​TGG​GAC​TTC​A-3).

## Results

### Prenatal course of the foetus

#### Pedigree 1

**FIGURE 1:**
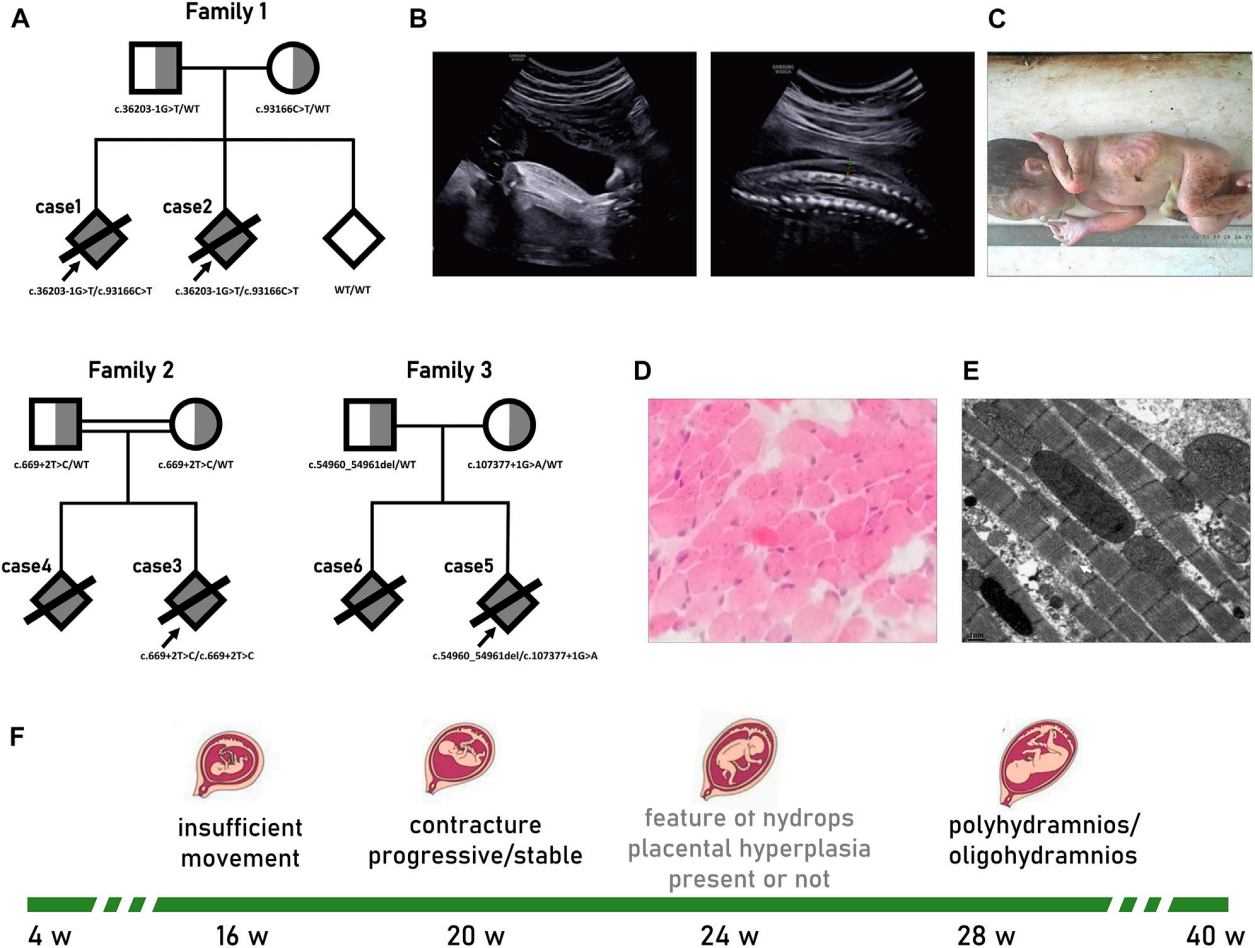
Pedigrees and prenatal phenotype of titinopathies. **(A)**Pedigrees of the fetus with Titinopathy profiled in this study. The proband is marked with arrow. **(B–E)** Results of II-2 from family 1. **(B)** Ultrasound at 28 weeks GA suggested bilateral talipes varus and skin edema. **(C)** Distal contracture characterized by ulnar deviation, flexion of phalanges; hypoplasia of fingers; and equinovarus feet was confirmed by autopsy. **(D)** Muscle tissues showed no obvious abnormality in histopathological pattern, while **(E)** ultrastructural lesions was deteced under electron microscope which manifested as myofibrillar disorganization, some of the sarcomeres disintegration. **(F)** Typical prenatal course of serve titin myopathy.

A 31-year-old female, G3P0, visited our centre at 11 weeks gestation age (GA) for genetic counselling due to two consecutive identical adverse pregnancies (cases 1 and 2) (pedigree in Figure 1A). The couple was not consanguineous, and both were in good physical condition. The wife accidentally detected slight tricuspid insufficiency with normal cardiac function in the detailed inspections.

According to the anamnesis, the first pregnancy (case 1) was complicated by an abnormal routine ultrasound scan at 20 w GA that showed a male foetus with poor movements of the lower limbs and right talipes. Six weeks later, the foetus manifested akinesia, bilateral clubfeet, pleural effusions, ascites, swollen liver and generalized skin oedema. Then, 1 week later, the face and cerebellum displayed blurry polyhydramnios. No anomalies were found in cardiac structure, placental blood flow, or middle cerebral artery peak systolic velocity (MCA-PSV). Non-immunologic hydrops foetalis (NIHF) due to aneuploidy, pathogenic copy number variation (pCNV), pathogen infection, haemoglobinopathies, blood group incompatibility, *etc.*, were excluded through the analysis of the amniotic fluid sample. Case 1 was stillborn at 30 w GA.

For case 2, routine ultrasound examination was unremarkable until 18 w GA, when the foetal movement was drastically reduced. At 22 w GA, the recurrence of bilateral varus and fixed posture was observed. The situation rapidly deteriorated over the next 6 weeks; we observed limited wrist extension, abnormal upper extremity posture, bilateral talipes varus, pericardial effusion, generalized skin oedema, multiple umbilical cord cysts, and mild polyhydramnios (Figure 1B). Case 2 was stillborn at 31 w GA. Autopsy confirmed distal arthrogryposis multiplex (Figure 1C). No macroscopic pathological changes were observed in the cardiac tissue except for a small amount of pericardial effusion. Muscle tissues displayed a normal histopathological pattern (Figure 1D), while ultrastructural lesions were detected under the electron microscope, such as myofibrillar disorganization and some of the sarcomere disintegration (Figure 1E).

#### Pedigree 2

A 24-year-old woman, G2P0, visited our centre at 16 w GA due to foetal megalocystis and unusual movement. Ultrasound at 19 w GA showed that urinary manifestations were exacerbated: megalocystis, lower urethral dilation, bilateral hydronephrosis, and a small amount of ascites. Furthermore, phenotypes that could not be explained by urethral obstruction were added: decreased movement, bilateral pes equinovarus, placental cyst, and placental thickening (55 mm). A further antenatal scan was organized at 24 w GA revealing a discrepancy in the foetal measurements with significant shortening of the limbs. At 29 w GA, polyhydramnios was observed. The woman finally decided to terminate the pregnancy. Autopsy confirmed the posterior urethral valves.

This couple are cousins, and the first pregnancy (case 4) was almost identical except for the signs of urinary tract obstruction. According to the medical record, the initial symptom was reduced movement at 15 w GA. Continuous ultrasound monitoring indicated that talipes equinovarus appeared 4 weeks later, and mild polyhydramnios was observed at 28 w GA. Then, the pregnancy was terminated without any other information.

#### Pedigree 3

A 33-year-old woman, G2P0, non-consanguineous marriage. The prenatal course (case 5) was unremarkable until ultrasound detected bilateral positional talipes equinovarus with reduced movement at 18 w GA. Reviewed after 8 weeks, foot contractures were almost non-progressive, but they showed disproportionate intrauterine growth. The amniotic fluid index slightly increased (27 cm) at 30 w GA, and the couple chose to terminate the pregnancy at 32 w GA.

According to the medical records, pedigree three had undergone the identical course for their first pregnancy (case 6) 2 years before. There were no signs of abnormal movement or abnormal posture until arthrogryposis presented at 17 w GA. For the next 10 weeks, the foetus had little progression of arthrogryposis but disproportionate limb growth with oligohydramnios and no signs of oedema. Pregnancy was terminated at 30 w GA.

### Genetic finding of the pedigrees

**FIGURE 2:**
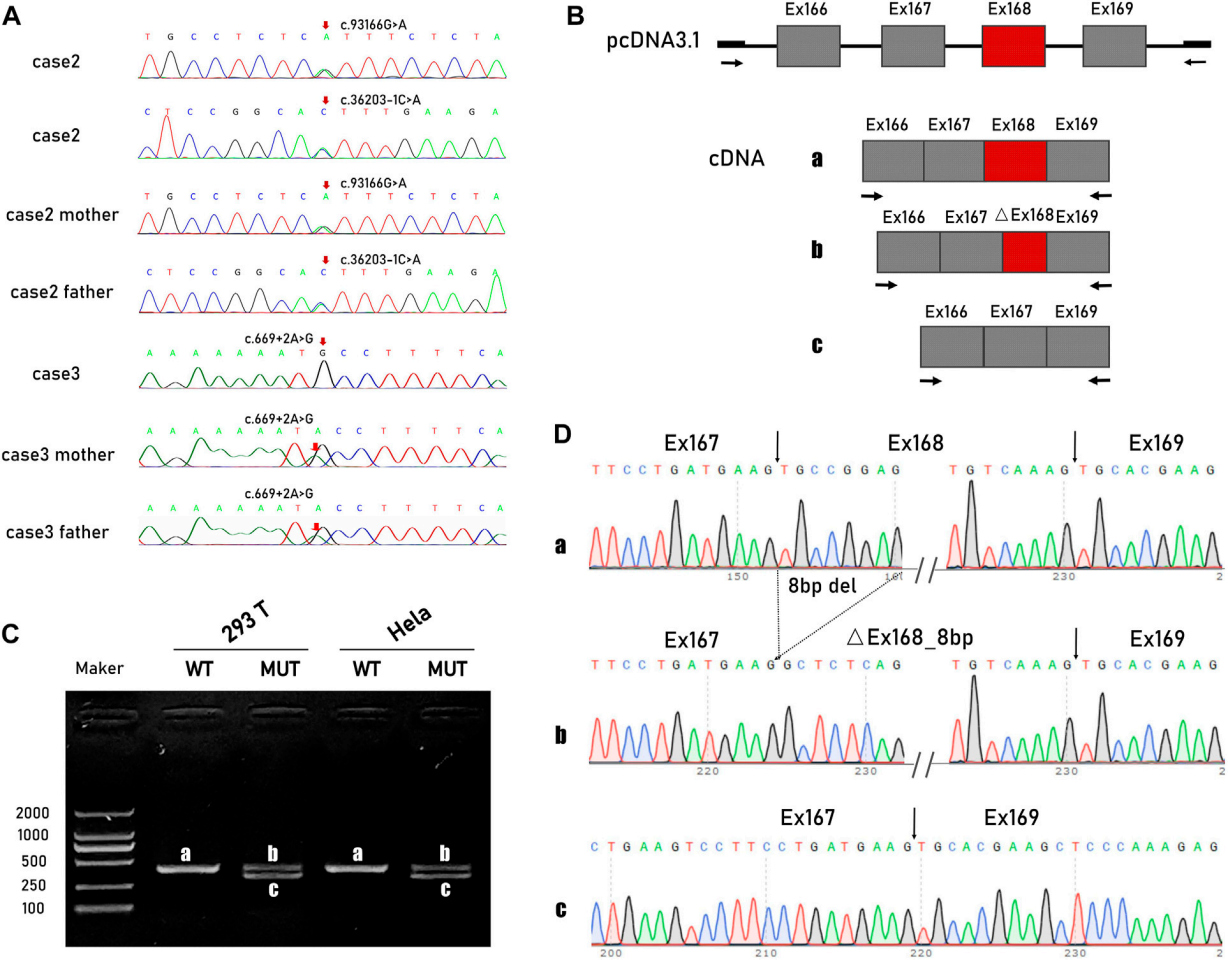
*TTN* variants and minigene studies of mRNA splicing following transient expression in Hela cells and HEK-293 cells. **(A)** Sanger sequencing of *TTN* variants in pedigree 1 and 2. **(B)** Schematic representation of the minigene vectors used for the *in vitro* splicing assay. **(C)** Electropherogram of PCR products. The wildtype control had the predicted size 408-bp band**(A)**, while the mutated had two smaller size 322-bp **(B)** and 330-bp band **(C)**. **(D)** Direct sequencing of the amplified minigene cDNA PCR products confirmed that the variant actives two different exonic cryptic acceptor sites with the subsequent activation of cryptic branch sites. Two altered transcripts were identified, one missing the last eight nucleotides of exon 167 and the second one skipping the exon 168.

*TTN* variants: c.93166C>T (*p*. Arg31056Ter) and c.36203-1G>T were detected in both case 1 and case 2. Other identified variants of uncertain significance (vus) that may be related to foetal hydrops were excluded by cross alignment (Supplementary Table S1). The maternally inherited non-sense variant c.93166C>T, in exon 339, affects all recognized postnatal transcripts apart from the short novex-3 striated muscle transcript. It has been reported in patients with end-stage DCM (Roberts et al., 2015). c.36203-1G>T is a meta-only transcript variant located in the I-band. Considering the existence of a large number of symmetrical repeating sequences in this region, a minigene was constructed to verify the transcriptional consequences of the splice site substitution (Figure 2B). cDNA analysis confirmed that the variant activated two different exonic cryptic acceptor sites with the subsequent activation of cryptic branch sites. Two altered transcripts were identified, one missing the last eight nucleotides of exon 167 and the second skipping exon 168 (Figures 2C, D).

A homozygous variant c.669 + 2 T>C was detected in case 3. The variant is evolutionarily conserved across multiple species and is not observed in ExAC or EVS. It is predicted to have an impact on exon six splicing (CADD scores 25.5, MutationTaster: Disease causing, varSEAK, HSF and MaxEnt predictions: damaging 100%).

In case 5, compound heterozygous variants were identified. c.54960_54961del is an A-band variant that introduces a frameshift and is predicted to create a premature stop codon. The splicing variant c.107377 + 1G>A is located between exon 362 and exon 363, which are the last two exons of the M-band. A cDNA study had already corroborated that the intronic variant caused a misplicing with two altered transcripts that missed the last 69 bp and the last seven bp of exon 362. Western blotting analyses have also shown a severe reduction in the C-terminal protein (Harris et al., 2017).

### Prenatal diagnosis for reproduction and follow-up

The woman from pedigree one underwent prenatal diagnosis at 12 weeks of gestation during her third pregnancy by chorionic villus sampling. Sanger sequencing showed that the foetus inherited neither allele (Figure 1A). Prenatal courses were uncomplicated. A female neonate 2,560 g in weight and 51 cm high was delivered full-term. Parameters of the infant development, strength and tension of muscle were all normal until the last visit at 12 months.

## Discussion

Few data are available on prenatal titinopathies because foetal phenotypes are non-specific and antenatal records are incomplete. It is estimated that 59% of affected foetuses exhibit insufficient movement, 17% show amniotic fluid changes in the third trimester, and only 7% manifest antenatal arthrogryposis (Oates et al., 2018). We described a series of titinopathy foetuses with different clinical severities and non-specific phenotypes that appeared in sequence, presenting certain characteristics. Recognizable features always begin with reduced foetal movement in the second trimester, followed by contracture, mainly distal. Hydrops, if they appear, usually occur 4–6 weeks later. Amniotic fluid changes (mostly polyhydramnio) always occur even later in the third trimester.

Hydrops in titinopathy are extremely rare but have special characteristics. The clinical course is different from the foetal akinesia (FA) deformation sequence caused by other pathogens. For variants in genes critical to excitation-contraction coupling (CACNA1S, SCN4A, STAC3), oedema may present as early as 15–18 w, simultaneously accompanied by arthrogryposis (Ravenscroft et al., 2021). For pathogenic variants in the gene encoding acetylcholine receptors (AGRN), in addition to the cooccurrence of oedema and contractures, the absence of oesophageal peristalsis also suggests the involvement of smooth muscle, which is not the target tissue of *TTN* variants (Geremek et al., 2020).

Thus, after excluding the common aetiology of oedema (such as immune, infectious, haematological, metabolic and lymphogenic) and considering the possibility of the involvement of the myocardium with *TTNtv*, we presumed that hydrops here is cardiogenic (Fomin et al., 2021). There is some evidence of histological deterioration to support our hypothesis. First, studies on isolated hearts found foetal hearts to be less compliant than adult hearts (Walker and de Tombe, 2004), which may be more sensitive to deleterious mutations. In a study on zebrafish, the mutant heart develops normally but is poorly contractile from the first beat and inevitably with oedema secondary to cardiac dysfunction (Xu et al., 2002; Santiago et al., 2021). Second, in foetuses presenting early-onset myopathy with fatal cardiomyopathy (EOMFC), which is a severe form of titinopathy, progressive dilated cardiomyopathy in early life also strongly suggests potential, primary defects in myocardial tissue (Carmignac et al., 2007). It could reasonably speculate an ultrastructural lesions had arised in myocardium, just as we have demonstrated in skeletal muscle.

We summarized all published congenital titinopathy cases who recorded typical prenatal phenotype, in anticipation of discovering patterns (Table 1). Almost all affected foetuses carry at least one pathogenic variant located in a metatranscript-only exon/intron. Biallelic *TTNtvs* in metatranscript-only exons, which are mainly scattered in the I-band, may be associated with severe foetal hydrops, contracturing, or even stillbirth. For the other 2 cases with typical contracture hydrops sequence reported to date (Table 1), metatranscript-only exon167 was involved (Chervinsky et al., 2018). Both of them were onset earlier under the background of homozygosity and experienced intrauterine/neonatal death. In here, c.36203-1G>T identified in cases 1/2 coincidentally generated an altered transcript that also affected exon 167. The recurrent occurrence of metatranscript-only exons 167, 168 in hydrops foetuses may suggest that as-yet-uncharacterized developmental isoforms, which implicate both myocardium and skeletal muscle containing these regions, are involved in the pathogenesis of this congenital disorder (Chervinsky et al., 2018; Oates et al., 2018).

**TABLE 1:** Summary of titinpathoy diagnosed prenatally.

Pedigree	Case		Nucleotide		Zygosity		Amino acid		Type		Exon		Region		Expressed in transcript														Prenata phenotype							Pregnancy outcome
															Meta		N2BA		N2A		N2B		Novex1		Novex2		Novex3		Movement		Contracture	Hydrops	IUGR	AF	Myocardial involvement	
Pedigree 4 Chervinsky et al., 2018	case 7		c.36122delC		homozygous		*p*.P12041Lfs*20		Framshift		exon167		I-band		√														Y		Y:at 24.1w no hands movements,flexion position of spine and legs	Y:at 24.1w edema around the feet	N	polyhydramnios at 27 weeks,AFI of 25 cm.	Suspected	died at 5w of respiratory failure
	case 8																												Y		Y:at 15w lower limbs and open hands placed constantly on the head. At 18 weeks the lower limbs were crossed and the upper limbs were flexed and remained in the same position.External examination showed fixed flexed position of knees and elbows, club feet,thin long bones and ribs	Y:at 20w	N	N	Suspected	intrauterine fetal death at 27.5w
Pedigree 1	case 1		c.36203-1G>T c.93166C>T		compound heterozygous		N/A *p*.Arg31056*		Splice site		intron167		I-band		√														UN		Y:at 20w	Y:at 26w	N	polyhydramnios at 26 weeks	Suspected	intrauterine fetal death at 30w
	case 2								Non-sense		exon339		A-band		√		√		√		√		√		√		√		Y:18w		Y:at 22w	Y:at 28w	N	polyhydramnios at 28 weeks	Suspected	intrauterine fetal death at 31w
Pedigree 2	case 3		c.669 + 2 T>C		homozygous		N/A		Splice site		Intron 5		Z-disk		√		√		√		√		√		√		√		Y:16w		Y:at 19w	N	N	polyhydramnios at 29 weeks	N	Top at 29w
	case 4																												Y:15w		Y:at 19w	N	N	polyhydramnios at 27 weeks	N	Top at 28w
Pedigree 3	Case5		c.54960_54961delc.107377 + 1G>A		compound heterozygous		*p*.Arg18321Serfs*3 N/A		Framshift		exon284		A-band		√		√		√										Y:18w		Y:at 18w	N	onset at 26 weeks	polyhydramnios at 26 weeks	N	TOP at 32w
	case 6								Splice site		intron362		M-band		√		√		√		√		√		√				Y17w		Y:at 17w	N	Onset 27weeks	oligohydramnios at 27 weeks	N	TOP at 30w
Pedigree 5 Bryen et al., 2020	case 9		c.38661_38665del		homozygous		*p*.Lys12887Asnfs*6		Framshift		exon197		I-band		√														Y in second trimester		Y in second trimester	N	UN	UN	N	caesarean-section at 36.2w
Pedigree 9 Bryen et al., 2020	case 10		c.39974–11 T>G c.28513G>T		compound heterozygous		N/A *p*.Glu9505*		Splice site		intron213		I-band		√														Y		N	N	N	N	N	hypotonia, axial weakness; finger/wrist/ankle/elbow/knee contractures; elongated face; micrognathia; low-set ears; died of respiratory failure at 2 years
	case 11								Non-sense		exon99		I-band		√														Y		N	N	N	N		hypotonia, axial weakness; finger/wrist/ankle/elbow/knee contractures; elongated face; micrognathia; low-set ears; severe restrictive lung disease
Pedigree 7 Bryen et al., 2020	case 12		c.39974–11 T>G c.37228delC		compound heterozygous		N/A *p*.Glu12411Lysfs*536		Non-sense		intron213		I-band		√														Y		Y: at 24w ulnar deviation, rocker Bottom feet	N	N	N	UN	TOP at 26w
									Framshift		exon180		I-band		√																					
Pedigree 8 Bryen et al., 2020	case 13		c.39974–11 T>G c.67279C>T		compound heterozygous		N/A *p*.Arg22427*		Splice site		intron213		I-band		√														Y		N	N	N	Oligohydramnious at 35 weeks	N	C-section at 35w, present axial weakness; finger/wrist/ankle/elbow/knee contractures; mild flat nasal bridge; respiratory involvement only at birth
									Non-sense		exon318		A-band		√		√		√		√		√		√											
Pedigree 10 McDermott et al., 2021	case 14		c.48681C>G c.104509_104510del		compound heterozygous		*p*.Tyr16227*p.Leu34837Glufs*12		Non-sense		exon260		A-band		√		√		√		√		√		√				Y		N	N	N	Polyhydramnios	UN	C-section at 36w, ostium secundum atrial heart defect, tricuspid valve dysplasia; died of respiratory failure at 2months
									Framshift		exon358		M-band		√		√		√		√		√		√											
Pedigree 6 McDermott et al., 2021	case 15		c.38415_38419del c.79750G>T		compound heterozygous		*p*.Lys12805Asnfs*45 Glu26584*		Framshift		exon194		I-band		√														Y		Y:elbows/knees/hip contractures	N	disproportionate intrauterine growth	polyhydramnios at third-trimester	N	axial hypotonia, arthrogryposis, respiratory insufficiency
									Non-sense		exon326		A-band		√		√		√		√		√		√											

IUGR:intrauterine growth restriction; AF:amniotic fluid, UN:unknown, N:no, Y:yes, w:week. a:sibling with similar symptoms died without exome sequencing, Gray block: fetus with arthrogryposis-hydrops sequence.

**FIGURE 3:**
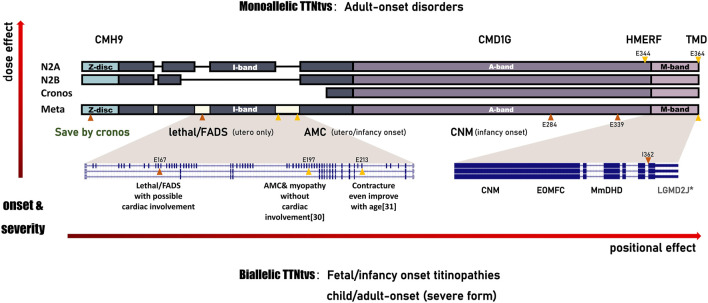
Schematic of the correlation between *TTNtv* and the phenotype spectrum of titinopathies. All the observed clinical features represent a continuum of phenotypes with an inverted relationship between the expression dose/position of truncation and the age of onset/clinical severity of these serial disorders. *TTNtvs* in the metatranscript-only area, which mainly distributed in I-band, result in FADS with/without cardiac involvement, or, even death. CMH9:familial hypertrophic cardiomyopathy-9. CMD1G:autosomal dominant dilated cardiomyopathy-1G.HMERF:hereditary myopathy with early respiratory failure. TMD:tibial muscular dystrophy. FADS:fetal akinesia deformation sequence. AMC:arthrogryposis multiplex congenita. CNM:centronuclear myopathy. EOMFC:early-onset myopathy with fatal cardiomyopathy. MmDHD:multi-minicore disease with heart disease. LGMD2J:limb-girdle muscular dystrophy type 2 J. Annotations include the following:variant reported here (orange triangles); variant reported in the literature (yellow triangles); asterisk (severe form of adult-onset).

Expanding our horizons to other foetal-onset cases, homozygous *TTNtv* in metatranscript-only exon197 results in arthrogryposis multiplex congenita and myopathy without cardiac involvement (Fernández-Marmiesse et al., 2017). In cases with a pathogenic *TTN* haplotype that includes the metatranscript-only exons 213–217, nearly 50% of congenital contractures improved with age (Bryen et al., 2020). Marco Savarese et al. speculated that it may be a slowly progressive disease for patients with at least one allele carrying a variant in metatranscript-only exons. (Savarese et al., 2020a). Further, they propose the concept of inverted relationship between the expression dose/position of truncation and the age of onset/clinical severity of recessive titinopathies in *postpartum* (Savarese et al., 2020a). Here, we supplemented the prenatal series, especially the rare hydrops-arthrogryposis subtype, on these cornerstones, and found that on the unique transcript of the fetus, the rule still holds and is more significant (Figure 3). For the dosage effect, patients harbouring monoallelic causative variants are prone to adult-onset myopathy with relatively mild symptoms or slow progression. Biallelic *TTNtvs* are always accompanied by a severe infantile/congenital phenotype. For the positional effect, clinical severity continues to increase from the M-band to the I-band. For example, a monoallelic variant in the final exon would induce a slowly progressive adult disease: tibial muscular dystrophy (TMD, 600334) (Hackman et al., 2002). Heterozygous *TTNtv* in the A-band-exon, which is constitutively expressed in the heart, is associated with an increased risk of DCM(604145). The age of onset is progressively earlier as the *TTNtvs* approach the N-terminal. Non-congenital cases always have at least one pathogenic variant in the final three exons (362–364) (De Cid et al., 2015; Savarese et al., 2020a). In case 5, variants involving exon 362 resulted in congenital, non-progressive arthrogryposis, indicating the potential effect of the second allele.

Paradoxically, the assumption of a continuum seemed to be invalid in case 3, who carried a homozygous *TTNtv* in the constitutively expressed Z-disc exon but had milder symptoms than expected. Carmignac V. et al. Proposed that homozygous recessive mutations within the N-terminal domain are lethal (Carmignac et al., 2007). It has been proven that titin Z-Disk truncations can be partially rescued by Cronos titin, although which produce lower contractile force and disarrayed sarcomeres (Zaunbrecher et al., 2019). Notably, it is predominantly a developmental isoform and is expressed at lower levels in early foetal skeletal muscle than in myocardium, which partly explains the presence of stable contractures without signs of cardiac involvement in case 3. Cronos titin has been shown to consistently decrease in intensity in cardiac samples as animals mature (Opitz et al., 2004). Lei Ye et al. demonstrated in a rat study that truncations of the *TTN* Z-disc predispose to heart failure with a preserved ejection phenotype in the context of pressure overload (Ye et al., 2018). So it is reasonable to speculate that truncation at the Z-disc will not have a significant effect on the myocardium prenatally, but the possibility of a gradual deterioration of cardiac function decades after birth cannot be ruled out.

## Conclusion

When suspected pathogenic TTN variants are detected or accidentally detected prenatally, it is challenging to accurately diagnose and counsel patients with inadequate phenotypic and limited pathological evidence. According to our study and summary, we recommend that the following points should be fully considered in prenatal diagnosis of Titinopathies.

1) When a foetus sequentially exhibits movement abnormalities, contracture, with/without hydrops, IUGR, and AF abnormalities, TTN-related FA should be considered and differentiated from neurogenic FA.
2) For the unexpected biallelic variation in *TTN* discovered by foetal NGS, the existence of a truncating background or in homozygosity may be a prerequisite for diagnosis (Savarese et al., 2020b). For the unexpected monoallelic *TTNtv*, long-term prognosis, which depends on the transcript, position, and penetrance, is the key point of consideration and consultation.
3) The downstream effect of splicing variants on the mRNA and protein levels in the proband’s muscles should be carefully evaluated whenever possible to minimize the chance that a causal relationship between the *TTN* variants and the clinical phenotype is misinterpreted.
4) Foetus with special meta-only transcript variants may be lethal, but postnatal contracture may be somewhat relieved with age if viable. Prognosis should consider the potential role of the second allele on the phenotype.

## Data Availability

The data presented in the study are deposited in the NCBI repository, accession number: PRJNA923837 (https://www.ncbi.nlm.nih.gov/sra/PRJNA923837).
